# Advancing Therapeutic Strategies in Atopic Dermatitis: Emerging Targets and Personalized Approaches

**DOI:** 10.3390/biom15060838

**Published:** 2025-06-08

**Authors:** Yang Lo, Ting-Ting Cheng, Chi-Jung Huang, Yu-Che Cheng, I-Tsu Chyuan

**Affiliations:** 1 Department of Dermatology, Cathay General Hospital, Taipei 010630, Taiwan; aicragyang@gmail.com (Y.L.);; 2 School of Medicine, College of Medicine, Fu Jen Catholic University, New Taipei 242062, Taiwan; yccheng@cgh.org.tw; 3 Department of Biochemistry, National Defense Medical Center, Taipei 114201, Taiwan; aaronhuang@cgh.org.tw; 4 Department of Medical Research, Cathay General Hospital, Taipei 106438, Taiwan; 5 Proteomics Laboratory, Department of Medical Research, Cathay General Hospital, Taipei 010630, Taiwan; 6 Department of Biomedical Sciences and Engineering, National Central University, Taoyuan City 320317, Taiwan; 7 School of Medicine, National Tsing Hua University, Hsinchu 030013, Taiwan; 8 Department of Internal Medicine, Cathay General Hospital, Taipei 010630, Taiwan

**Keywords:** atopic dermatitis, management, thymic stromal lymphopoietin, OX40, IL-22, IL-17, IL-31, histamine receptor, skin microbiome

## Abstract

Atopic dermatitis (AD) is a chronic inflammatory skin disorder marked by intricate interplay among skin barrier dysfunction, immune dysregulation, and microbial dysbiosis. While therapeutic advancements targeting T helper 2 (Th2) cytokines, such as interleukin (IL)-4 and IL-13, and the Janus kinase/signal transducer and activator of transcription (JAK/STAT) pathway have yielded promising outcomes, a significant proportion of patients still experience inadequate relief, particularly from persistent pruritus. Achieving minimal disease activity remains an unmet clinical priority and a cornerstone of effective AD management. This review provides an in-depth analysis of current therapeutic approaches and integrates findings from recent biologic studies, with a particular focus on innovative strategies under active investigation. These approaches include targeting components of the innate immune system, such as thymic stromal lymphopoietin (TSLP) and IL-1 family cytokines; the adaptive immune system, including OX40-OX40L interactions and Th17- and Th22-related cytokines; and mechanisms associated with pruritus, such as IL-31, histamine receptors, and neurokinin 1 receptor. Emerging insights underscore the transformative potential of personalized therapeutic regimens tailored to the distinct endotypes and severity of AD. Advances in deciphering the pathogenesis of AD are unlocking unprecedented opportunities for precision medicine, offering renewed hope for improved outcomes in this multifaceted and heterogeneous condition.

## 1. Introduction

**Figure 1:**
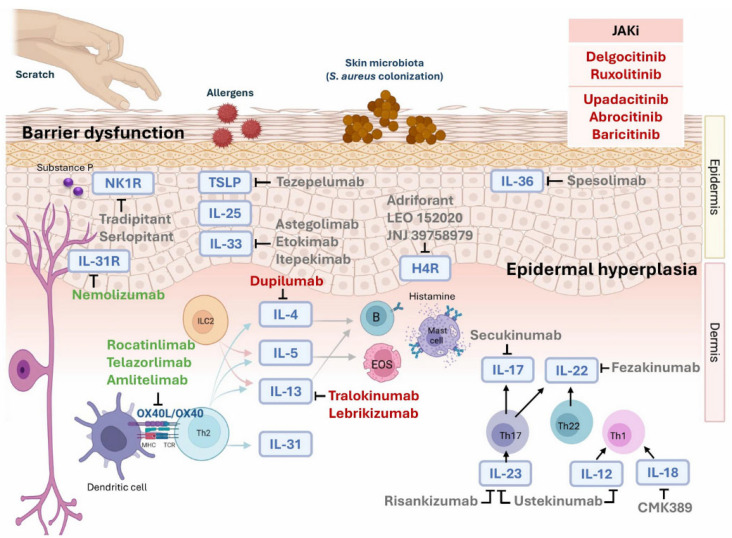
Therapeutic targets in atopic dermatitis: Novel treatments depicted in gray have failed to demonstrate efficacy in phase 2 or phase 3 trials. Promising agents, such as nemolizumab and OX40/OX40L inhibitors, are depicted in green. Approved agents, like dupilumab, tralokinumab, lebrikizumab, and JAK inhibitors, are depicted in red. Abbreviations: *H4R*, Histamine 4 Receptor; *JAKi*, Janus kinase inhibitors; *NK1R*, neurokinin 1 receptor; *TSLP*, thymic stromal lymphopoietin.

Atopic dermatitis (AD) is a chronic inflammatory disorder caused by a complex interplay of genetic susceptibility, environmental factors, skin barrier dysfunction, microbial imbalances, and immune dysregulation. During the acute phase, damaged keratinocytes release alarmins such as interleukin (IL)-25, IL-33, and thymic stromal lymphopoietin (TSLP) in response to environmental insults or tissue injury, leading to the activation of the Th2 immune response. This immune activation results in increased levels of key cytokines, particularly IL-4, IL-5, and IL-13. As AD progresses to a chronic state, immune responses gradually shift toward Th1 and Th17 pathways (Figure 1) [1,2]. Advances in research have substantially improved our understanding of AD pathogenesis. Co-stimulatory molecules such as OX40 and its ligand OX40L may promote the activation, proliferation, and survival of pathogenic T cells [3]. In addition to Th2-driven pathways, Th22 cells have been implicated in AD [4]. Dysbiosis of the skin and gut microbiomes may exacerbate AD symptoms and modulate immune responses. Consequently, emerging therapeutic strategies are increasingly aimed at targeting key components of AD pathogenesis. This review first outlines the approved treatments currently used in clinical practice, then presents the latest insights into AD pathogenesis, as well as potential therapeutic approaches currently under investigation.

## 2. Topical Treatments for AD

Beyond traditional topical treatments like corticosteroids, calcineurin inhibitors, and PDE-4 inhibitors, topical JAK inhibitors have emerged as an alternative for managing mild to moderate AD. Ruxolitinib 1.5% cream, a selective JAK 1/2 inhibitor, is approved for short-term and intermittent chronic use in patients aged ≥ 12 years with mild to moderate AD [5]. In phase 3 trials, 51.3% to 53.8% of patients achieved treatment success by week 8, with notable itch reduction occurring within 12 h of application [6]. The frequently reported side effects included upper respiratory tract infection and nasopharyngitis [7]. Delgocitinib, a pan-JAK inhibitor is approved for the management of both pediatric patients (≥2 years) and adults with AD in Japan, showing a 39.3% to 44.3% reduction in the modified eczema area and severity index (EASI) score by week 4 [8,9]. These findings highlight the growing role of topical JAK inhibitors as effective and well-tolerated options for both pediatric and adult patients with mild to moderate AD.

## 3. Systemic Treatments for AD

### 3.1. Biologics and Small Molecule Inhibitors for AD

Novel biologics and small-molecule inhibitors have revolutionized AD treatment since 2017. Dupilumab, the first biologic approved by the FDA, inhibits IL-4 and IL-13 by targeting the alpha subunit of the IL-4 receptor. Tralokinumab, the second FDA-approved biologic, blocks IL-13 by inhibiting its binding to receptor chains IL-13Rα1 and IL-13Rα2. Lebrikizumab, another newly approved IL-13-targeting monoclonal antibody, inhibits the IL-4Rα–IL-13Rα1 signaling pathway, while still enabling the regulation of IL-13 activity via the IL-13Rα2 receptor. Additionally, the FDA approved two oral JAK inhibitors, upadacitinib and abrocitinib, which selectively inhibit the JAK-1 pathway. Baricitinib, a JAK-1/2 inhibitor, is approved in Europe for AD management, though it has not yet received FDA approval. These biologics and small-molecule inhibitors represent a significant advancement in the treatment of AD, offering targeted therapies that directly address the underlying immune dysregulation, leading to improved outcomes.

A network meta-analysis of randomized clinical trials assessing the short-term efficacy (up to 16 weeks) in moderate-to-severe atopic dermatitis found that upadacitinib (30 mg daily) and abrocitinib (200 mg daily) and provided better improvements in key clinical measures compared to dupilumab (600 mg initially, then 300 mg every two weeks). These measures included the EASI, patient-oriented eczema measure (POEM), dermatology life quality index (DLQI), and peak pruritus numerical rating scale (PP-NRS). Upadacitinib at a lower dose (15 mg daily) showed similar effectiveness to dupilumab. On the other hand, abrocitinib (100 mg daily), baricitinib (2 mg and 4 mg daily), tralokinumab (600 mg initially, then 300 mg every two weeks), and lebrikizumab (500 mg at weeks 0 and 2 initially, then 250 mg every two weeks) were linked to lower improvements in these clinical outcomes [10,11].

These findings are increasingly supported by real-world evidence. Recent observational studies have confirmed the effectiveness and safety of both biologics and JAK inhibitors in routine clinical settings. Among the JAK inhibitors, upadacitinib demonstrated the most robust outcomes, with approximately 60% achieving EASI 90 at 3 months in the UP-TAINED study and 67.7% reaching EASI 90 at week 16 in another single-center real-world study [12,13]. Sustained disease control for up to one year was also observed, including in difficult-to-treat areas such as the hands and face [12,13,14]. Notably, even patients who did not achieve EASI 75 by week 12 showed gradual and continued improvement over time, highlighting the potential long-term benefits of upadacitinib therapy [14]. Similarly, abrocitinib showed promising results, with 50.5% of patients reaching EASI 90 at 3 months in a prospective single-center study conducted in China [15]. In contrast, biologics showed more modest response rates in real-world settings, with EASI 90 rates at 16 weeks of 26.8% for dupilumab, 26% for tralokinumab, and 27.3% for lebrikizumab, respectively [16,17,18].

### 3.2. Therapies Under Investigation

#### 3.2.1. TSLP and the IL-1 Family

In AD, skin barrier dysfunction facilitates foreign antigens to penetrate. Upon allergen exposure, keratinocytes release alarmins such as IL-25, IL-33, and TSLP, which play vital roles in AD. These alarmins increase the extent of type 2 inflammation and suppress the expression of barrier-associated proteins such as loricrin, filaggrin, and involucrin, further compromising skin barrier integrity. TSLP activates dendritic cells, leading to the production of Th2-attracting chemokines (CCL17 and CCL22) and promoting the differentiation of naïve CD4+ T cells into Th2 cells [19]. Studies involving transgenic mice have demonstrated that TSLP overexpression in keratinocytes induces AD-like skin inflammation [20]. TSLP is highly expressed in the epidermis of both acute and chronic AD lesions. Its level is elevated also in serum,, correlating positively with disease severity [19,21]. Although preclinical studies have implicated TSLP in AD pathogenesis, tezepelumab—an anti-TSLP monoclonal antibody approved as an add-on therapy for severe asthma—conferred no substantial clinical benefits over placebo in phase 2 trials for AD (Table 1) [22]. Concomitant use of topical corticosteroids (TCS), particularly high-dose potent TCSs in the placebo group, as well as the 12-week treatment period, might have affected the outcome of the aforementioned trial, given the upstream positioning of TSLP in the inflammatory cascade [22].

The IL-1 cytokines family is integral to the innate immune system and may initiate inflammation in AD. This family comprises four major subfamilies—IL-1, IL-18, IL-33, and IL-36—most of which are well-known proinflammatory cytokines [23]. IL-18 activates basophils and mast cells, promoting the production of histamine, IL-4, and IL-13. It also enhances both Th2 and Th1 immune responses, with IL-12 and IL-18 together driving a shift toward Th1/0 responses that contribute to the chronic phase of AD [24]. IL-33 activates various immune cells, particularly type 2 innate lymphoid cells, stimulating the release of type 2 cytokines. It also contributes to pruritus itch by inducing IL-31 production and impairs skin barrier integrity by downregulating the production of filaggrin and claudin-1 [25]. IL-36α, produced by keratinocytes in response to *Staphylococcus aureus* infection, further upregulates IL-17 expression. Intradermal injection of IL-36α has been reported to induce acanthosis and leukocyte infiltration in the murine skin. IL-36 is also implicated in the transition of AD from the acute phase to the chronic [26]. Experimental studies have demonstrated that skin-specific expression of IL-1 family cytokines can induce AD-like eczema in mice, and that these cytokines are considerably upregulated in both serum and lesional skin of patients with AD [24,25,27].

Several biologics targeting IL-1 family cytokines have been evaluated for AD, but many have failed to exhibit clinical efficacy. Although the anti-IL-1α monoclonal antibody (bermekimab), and the anti-IL-33 monoclonal antibodies (astegolimab, etokimab, and itepekimab) were developed for AD treatment, they were either terminated or found to confer no substantial benefits in phase 2 trials [28,29,30,31] (Table 1). Similarly, spesolimab, an IL-36 receptor antagonist approved for generalized pustular psoriasis, is no longer under evaluation for AD. A phase 2a trial reported only modest effects compared with placebo, suggesting that IL-36 plays a secondary role in AD pathogenesis [32] (Table 1). Preclinical data indicate that IL-18 deficiency reduces AD-like skin lesions in mice [33]. Currently, a phase 2 clinical trial (NCT04836858) is investigatingCMK-389, an IL-18 inhibitor, in patients with moderate-to-severe AD. By week 16, 11.8% to 14.7% of all patients receiving CMK-389 achieved an Investigator’s Global Assessment (IGA) response, defined as clear or almost clear skin with a minimum 2-point reduction from baseline [34].

**Table 1:** Clinical trials of novel treatments targeting innate immune system for atopic dermatitis.

Target	Medication	Study Design	Primary Outcome	Clinical Trial	Status of Investigation
TSLP	Tezepelumab	113 adults with moderate to severe AD	EASI50 at Week 12 Tezepelumab+TCS: 64.7% Placebo+TCS: 48.2% Odds Ratio: 1.97 (95% CI: 0.90 to 4.33)	NCT02525094 (Phase II) [22]	Complete, but failed to meet efficacy
IL-1α	Bermekimab	87 adults with moderate to severe AD	EASI75 at Week 16 Bermekimab 400 mg qw: 34.5% (*p* = 0.04) Bermekimab 400 mg q2w: 24.1% (*p* = 0.35) Placebo: 13.8%	NCT04021862 (Phase II) [30]	Complete, but failed to meet efficacy
		130 adults with moderate to severe AD	EASI75 at Week 16 Bermekimab 350 mg qw: 16.7% (*p* = 0.489) Bermekimab 700 mg q2w: 16.7% (*p* = 0.448) Placebo: 9.5%	NCT04021862 (Phase II) [30]	Terminated due to meeting futility criterion
IL-18	CMK389	71 adults with moderate to severe AD	IGA clear or almost clear and at least a 2-grade improvement from baseline at Week 16 CMK389 10 mg/kg monthly: 14.7% CMK389 300 mg monthly: 11.8% Placebo: 0%	NCT04836858 (Phase II) [34]	Complete
IL-33	Astegolimab	65 adults with moderate to severe AD	% change in the EASI score at Week 16 Astegolimab loading 245 mg/490 mg q4w: −51.47 (8.639) Placebo: −58.24 (9.092) Mean (Standard Error)	NCT03747575 (Phase II) [28]	Complete, but failed to meet efficacy
	Etokimab	302 adults with moderate to severe AD	% change in the EASI score at Week 16 Etokimab 20 mg q4w: −41.63 (6.707) Etokimab loading 300 mg/150 mg q8w: −55.70 (6.21) Etokimab loading 300 mg/150 mg q4w: −47.40 (6.09) Etokimab loading 600 mg/300 mg q4w: −44.56 (7.81) Placebo: −49.38 (7.12) Mean (Standard Error)	NCT03533751 (Phase II) [35]	Complete, but failed to meet efficacy
	Itepekimab	206 adults with moderate to severe AD	% change in the EASI score at Week 16 Itepekimab 300 mg q2w: −66.6 (22.46) Placebo: −52.4 (31.86) Mean (Standard Error)	NCT03736967 (Phase II) [31]	Terminated due to meeting futility criterion
IL36R	Spesolimab	51 adults with moderate to severe AD	% change in the EASI score at Week 16 Spesolimab 600 mg q4w: −37.9 (9.8) Placebo: −12.3 (14.3) (*p* = 0.149) Mean (Standard Error)	NCT03822832 (Phase II) [32]	Complete, but failed to meet efficacy
		14 adults with moderate to severe AD	Number of patients with treatment emergent adverse events at week 48: 9/14 (64.3%)	NCT04086121 (Phase II) Open Label Extension Study of NCT03822832 [36]	Terminated

#### 3.2.2. OX40 and OX40L

OX40 (CD134) and its ligand OX40L (CD252) function as secondary co-stimulatory molecules that enhance T cell responses [37]. After antigen exposure, naïve T cells are initially activated by antigen-presenting cells (APCs) through primary co-stimulatory signals provided by the CD40/CD40L and B7/CD28 pathways. Within 24 h of activation, OX40L is expressed on APCs such as dendritic cells, Langerhans cells, type 2 innate lymphoid cells, and mast cells. OX40 is subsequently expressed on activated T cells, typically within 1 to 5 days [3,38]. The interaction between OX40 and OX40L activates the nuclear factor-κB1 and phosphoinositide 3-kinase/protein kinase B pathways, promoting the proliferation, differentiation, and survival of effector T cells. These signaling cascades also supports the generation and reactivation of memory T cells, which are essential for long-term immune responses [3,37]. OX40 signaling may inhibit the function of regulatory T cells by downregulating FOXP3 expression, thereby enhancing effector T cell activity by mitigating immunosuppression [3,37].

In AD, allergen exposure triggers the release of alarmins, enhancing OX40L expression on APCs. The subsequent interaction between OX40 and OX40L promotes Th2-dominant signaling and T-cell differentiation, leading to the production of type 2 inflammatory cytokines and facilitating the development of early acute stage AD lesions [39]. As the condition progresses to a chronic phase, sustained OX40-OX40L signaling drives the proliferation of effector T cell populations, such as Th1, Th17, and Th22 cells. These cells release proinflammatory cytokines such as interferon-gamma, IL-17, and IL-22, which intensify inflammation and promote keratinocyte proliferation and epidermal thickening. Persistent activation of these T cell populations through OX40 signaling establishes an inflammatory cycle reinforcing the chronic nature of AD [38,39].

A study demonstrated that lesional skin from patients with AD exhibited a markedly higher intensity of OX40L staining and a higher number of OX40-positive cells in the dermis than did non-lesional skin [40]. Furthermore, OX40 expression was upregulated on skin-homing T cells in the serum of patients with AD, indicating that the OX40 axis is associated with both local and systemic involvement in AD [41]. Tape-strip analyses confirmed the upregulation of the OX40L/OX40 immune pathway in lesional AD skin compared with the findings in healthy skin [42,43]. Several experimental drugs targeting this pathway are currently in development; for example, anti-OX40 drugs such as rocatinlimab (AMG 451/KHK4083) and telazorlimab (ISB 830/GBR 830), as well as anti-OX40L antibodies such as amlitelimab (KY1005/SAR445229) (Table 2).

Rocatinlimab (anti-OX40 antibody) outperformed the placebo after a 16-week treatment in a phase 2b trial involving 274 patients with moderate-to-severe AD. Across multiple dosing groups, least squares mean percent reductions in EASI scores ranged from 48 to 61%, while only 15% reduction was observed in the placebo group. A 300 mg biweekly (Q2W) dose yielded the most pronounced response, with 54% of patients achieving EASI75, and 31% achieving an IGA score of 0 or 1 by week 16 [44]. Rocatinlimab also exhibited potential for sustained efficacy: at week 36, patients receiving the 300 mg Q2W dose exhibited an 88% reduction in the EASI scores, with 64% achieving EASI75 and 52% attaining an IGA score of 0 or 1. In addition, rocatinlimab substantially modulated AD-associated biomarkers, reducing serum levels of thymus and activation-regulated chemokine/chemokine (C-C motif) ligand (CCL)17 (a Th2 marker) and IL-22 (associated with Th17/Th22 [44]. The treatment was well tolerated, with the predominant adverse events including pyrexia (17% vs. 4% in the placebo group), chills (11% vs. 0%), headache (9% vs. 2%), aphthous ulcers (7% vs. 0%), and nausea (6% vs. 2%) [44]. Rocatinlimab is currently being evaluated in phase 3 trials (NCT05633355, NCT05651711, NCT05398445, NCT05899816, NCT05724199, NCT05882877, NCT05704738).

In an exploratory phase 2a study, telazorlimab (GBR 830) (anti-OX40 antibody) was well-tolerated when administered in two doses spaced 4 weeks apart [45]. The treatment led to marked reductions in the mRNA expression levels of Th1 markers (IFN-γ/CXCL10), Th2 markers (IL-31/CCL11/CCL17), and Th17/Th22 markers (IL-23p19/IL-8/S100A12) in lesional skin. Improvements were observed in markers of epidermal hyperplasia (skin thickness, keratin 16, and Ki67) [45]. Clinical and histological improvements progressed through week 10 (6 weeks after the final dose). By that time, 76.9% (20/26) of all patients in the telazorlimab group achieved EASI50, while 37.5% (3/8) of all patients in the placebo group achieved EASI50 [45]. In a phase 2b trial, higher doses of telazorlimab (600 mg loading, then 300 mg Q2W, and 1200 mg loading, then 600 mg Q2W) led to considerable reductions in the EASI score compared with the outcomes of placebo treatment (−54.4% vs. −34.2% and −59.0% vs. −41.8%, respectively; *p* = 0.008 for both) [46]. However, no improvement in pruritus was noted at week 16 compared with baseline condition. Safety outcomes were similar between the treatment and placebo groups, with the most common adverse events being AD exacerbation, nasopharyngitis, upper respiratory tract infections, and headache. Currently, no phase 3 trials are underway for telazorlimab [46].

Amlitelimab, a noncytotoxic anti-OX40L IgG4 monoclonal antibody, has proven effective in a phase 2b clinical trial for AD. Marked reductions in the EASI score (ranging from −51.6% to −61.5%) were observed at week 16 across multiple dose groups compared with the findings in the placebo group. Improvements were also noted in key secondary outcomes, such as EASI75, IGA 0/1, and PP-NRS ≥ 4. Notably, sustained efficacy (defined as an IGA score of 0 or 1and/or EASI75) maintained through week 52, even in patients who had discontinued treatment or exhibited low to negligible serum drug concentrations [47]. Amlitelimab is currently advancing into phase 3 trials in adults with moderate-to-severe AD (NCT06181435 and NCT06130566).

**Table 2:** Clinical trials of novel treatments targeting the adaptive immune system for atopic dermatitis.

Target	Medication	Study Design	Primary Outcome	Clinical Trial	Status of Investigation
OX40L	Amlitelimab	89 adults with moderate to severe AD	% change in the EASI score at Week 16 Amlitelimab loading 200 mg/100 mg q4w: −80.12% (95% CI: −95.55 to −64.68) (*p* = 0.009) Amlitelimab loading 500 mg/250 mg q4w: −69.97% (95% CI: −85.04 to −54.60) (*p* = 0.07) Placebo: −49.37% (95% CI: −66.02 to −32.72)	NCT03754309 (Phase II) [48]	Complete
		390 adults with moderate to severe AD	% change in the EASI score at Week 16 Amlitelimab loading 500 mg/250 mg q4w: −61.5% (95% CI: −43.9 to −20.3) (*p* < 0.0001) Amlitelimab 250 mg q4w: −56.8% (95% CI: −39.1 to −15.6) (*p* < 0.0001) Amlitelimab 125 mg q4w: −51.6% (95% CI: −34.0 to −10.4) (*p* = 0.0002) Amlitelimab 62.5 mg q4w: −59.6% (95% CI: −41.9 to −18.5) (*p* < 0.0001) Placebo: −29.4%	NCT05131477 (Phase II) [47]	Complete
OX40	Rocatinlimab (AMG 451/KHK4083)	274 adults with moderate to severe AD	% change in the EASI score at Week 16 Rocatinlimab 150 mg q4w: −48.33 (95% CI: −62.62 to −34.04) Rocatinlimab 600 mg q4w: −49.72 (95% CI: −62.47 to −35.17) Rocatinlimab 300 mg q2w: −61.07 (95% CI: −75.19 to −46.96) Rocatinlimab 600 mg q2w: −57.35 (95% CI: −71.27 to −43.43) Placebo: −15.01 (95% CI: −28.60 to −1.43) Statistically significant improvements in all groups	NCT03703102 (Phase II) [44]	Complete
OX40	Telazorlimab (ISB 830/ GBR 830)	64 adults with moderate to severe AD	Change from baseline in lesional epidermal thickness at week10 Telazorlimab 10 mg/kg q4w: −26.51 (88.68) Placebo: −6.01 (39.40)	NCT02683928 (Phase II) [45]	Complete
		462 adults with moderate to severe AD	% change in the EASI score at Week 16; Mean (Standard Error) Telazorlimab loading 600 mg/300 mg q2w: −54.4 (5.1) (*p* = 0.008) Telazorlimab loading 600 mg/300 mg q4w: −48.6 (5.4) (*p* = 0.06) Telazorlimab loading 150 mg/75 mg q4w: −31.0 (5.7) (*p* = 0.69) Placebo 2 injections q2w: −34.2 (5.5) Telazorlimab loading 1200 mg/600 mg q2w: −59.0 (4.6) (*p* = 0.008) Placebo 4 injections q2w: −41.8 (4.7)	NCT03568162 (Phase II) [46]	Complete
IL-12/ IL-23	Ustekinumab	79 Japanese adults with severe AD	% change in the EASI score at Week 12; Mean (Standard Error) Ustekinumab 45 mg at Week 0 and 4: −38.62 (32.684) Ustekinumab 90 mg at Week 0 and 4: −39.39 (38.710) Placebo: −37.54 (37.592) Both treatment arms allowed concurrent use of TCS, topical calcineurin inhibitors, or anti-leukotriene therapies	NCT01945086 (Phase II) [49]	Complete, but failed to meet efficacy
		32 adults with moderate to severe AD	SCORAD50 at Week 16 Ustekinumab+TCS: 31.3%; Placebo+TCS: 18.8% Odds Ratio: 1.93 (95%CI: 0.30 to 15.33)	NCT01806662 (Phase II) [50]	Complete, but failed to meet efficacy
IL-23	Risankizumab	172 adults and adolescents with moderate to severe AD	EASI75 at Week 16 Risankizumab 150 mg at Week 0 and 4: 24.6% (95%CI: 14.5 to 34.8) (*p* = 0.08) Risankizumab 300 mg at Week 0 and 4: 21.7% (95%CI: 12.0 to 31.5) (*p* = 0.18) Placebo: 11.8% (95%CI: 0.9–22.6)	NCT03706040 (Phase II) [51]	Complete, but failed to meet efficacy
IL-17	Secukinumab	41 adults with moderate to severe AD	Fold change in epidermal thickness of lesional skin at Week 16 Secukinumab only for extrinsic AD: 1.18 (1.15) Secukinumab only for intrinsic AD: −1 (1.15) Placebo then secukinumab from week 16 for extrinsic AD: 1.15 (1.22) Placebo then secukinumab from week 16 for intrinsic AD: 1.5 (1.3)	NCT02594098 (Phase II) [52]	Complete, but failed to meet efficacy
IL-22	Fezakinumab (ILV-094)	60 adults with moderate to severe AD	% change in the SCORAD at Week 12 Fezakinumab loading 600 mg/300 mg q2w: −13.8 (2.7) Placebo: −8 (3.1) (*p* = 0·134) % change in the SCORAD at Week 20 Fezakinumab loading 600 mg/300 mg q2w: −18.8 (2.9) Placebo: −11.7 (3.9) (*p* = 0·049)	NCT01941537 (Phase II) [53]	Complete

#### 3.2.3. IL-17 and Th17-Related Cytokines

Beyond clinical phenotype, defining AD endotypes at the cellular and molecular levels has become crucial for developing personalized, targeted treatment strategies. Although no universal classification strategy has been established, several studies have stratified patients on the basis of clinicodemographic characteristics such as ethnicity (e.g., African, Asian, European), intrinsic versus extrinsic disease (based on serum IgE levels), and age (children, adults, and older adults). These categorizations facilitate comparisons of cytokine expression patterns, skin barrier dysfunction, and serum biomarker profiles [54]. In extrinsic AD, elevated IgE levels indicate a predominantly Th2-driven inflammatory response. By contrast, intrinsic AD is characterized by normal IgE levels, upregulated IFN-γ, IL-17, and IL22 expression, and increased Th2 activity. In patients with intrinsic AD, Th17 markers are positively correlated with SCORAD scores [55]. Ethnic variations further highlight the complexity of AD endotypes. The Asian AD phenotype presents the immune features of both AD and psoriasis, for example, parakeratosis, increased epidermal hyperplasia, enhanced Th17 activation, and Th2 activation levels similar to those observed in European American patients with AD [56,57]. Age-related differences also contribute to immune heterogeneity in AD. Lesions from pediatric patients exhibit elevated IFN-γ and Th17 responses, whereas non-lesional skin exhibit increased Th17 and Th22 responses. Infants also exhibit higher regulatory T cells levels and greater Th17 activity than adults. Pediatric AD is characterized by prominent Th17/Th22 skewing, but lacks the Th1 upregulation noted in adults [58]. These findings suggest that targeting the Th17 pathway is a promising therapeutic strategy for intrinsic AD, pediatric AD, and the Asian AD phenotype.

Several Th17-targeting biologics, which were originally approved for psoriasis, have been evaluated for AD; however, these agents exhibited insufficient clinical efficacy (Table 2). Ustekinumab, which inhibits IL-12 and IL-23, and thereby suppresses downstream cytokines such as IL-17 and IL-22, was evaluated in a phase 2 trial with Caucasian patients with moderate-to-severe AD. Although no significant clinical improvements were observed, genomic profiling at week 4 revealed marked downregulation of Th1-, Th17-, Th22-, and Th2-related genes in the ustekinumab group [50]. Similarly, a phase 2 trial involving Asian patients with moderate to severe AD reported no significant reduction in the EASI score in the ustekinumab group compared with the findings in the placebo group [49]. The concurrent use of TCSs in both studies might have reduced the observable differences between the treatment and placebo groups, yielding non-significant results. In a phase 2 trial of secukinumab, an IL-17A inhibitor, no significant differences in epidermal thickness or clinical outcomes, as measured using the EASI and SCORAD tools, were observed between the secukinumab and placebo groups. Neither group exhibited meaningful clinical improvement compared with the baseline. These results remained consistent even after subgroup analyses by AD type (intrinsic vs. extrinsic AD) and in a post hoc analysis of Asian patients with AD [52]. Together, the findings indicate that targeting Th17-related cytokines alone is insufficient for treating AD, even in patients with an enhanced Th17 signature.

#### 3.2.4. IL-22

Elevated levels of IL-22 have been detected in both the serum and skin biopsies of patients with chronic AD [59,60]. IL-22, primarily produced by Th17 and Th22 cells, contributes to epidermal hyperplasia by promoting keratinocyte proliferation and disrupting the skin barrier through the suppression of proteins essential for keratinocyte differentiation [61]. In addition, IL-22 upregulates proinflammatory cytokines, such as S100A7, S100A8, and S100A9, which facilitate the recruitment of immune cells [4]. It also strongly induces the expression of gastrin-releasing peptide (GRP), a neuropeptide pruritogen, in sensory afferents and dermal immune cells, and upregulates that of its receptor on keratinocytes, thereby contributing to pruritus in AD [62]. Fezakinumab (FZ, ILV-094), an anti-IL-22 antibody, was assessed in a phase 2a clinical trial involving 60 patients with moderate to severe AD (Table 2). Participants received an initial intravenous dose of 600 mg of FZ, followed by 300 mg every 2 weeks for 10 weeks, and monitored for subsequent 20-weeks. Although the primary outcome—change in SCORAD score from baseline—was nonsignificant at week 12, statistical significance was reached by week 20. Notably, patients with severe AD (SCORAD ≥ 50) exhibited significant improvement at week 6 [53]. Skin biopsies demonstrated that FZ effectively reversed AD-associated gene expression profiles, particularly in patients with high baseline IL-22 levels, who also exhibited considerable transcriptomic improvements. Treatment reduced the expression of genes involved in inflammation, T-cell activation, and immune responses, but increased that of the negative immune regulators, IL-34 and IL-37 [63]. These findings suggest that FZ is particularly effective in patients with severe AD and high baseline IL-22 levels.

## 4. Special Consideration for AD

### 4.1. Modulation of the Skin Microbiome

Dysbiosis of the cutaneous microbiota in AD may exacerbate skin barrier dysfunction, increasing infection risks, intensifying inflammation, and worsening disease symptoms. AD is characterized by a marked reduction in microbial diversity, with an overrepresentation of pathogenic bacteria, particularly *S. aureu*s, and a corresponding decline in beneficial commensals such as *Cutibacterium* species [64,65]. *S. aureus* produces virulence factors that lead to epidermal damage and stimulate the release of proinflammatory cytokines [66]. Its capacity to form biofilms further enhances bacterial adhesion, shields against antimicrobial agents, and facilitates persistent colonization, which is associated with severe AD [67,68].

Traditionally, the treatment of *S. aureus* over-colonization in AD has involved both indirect and direct strategies. Indirect strategies include reducing skin pH with emollients to inhibit *S. aureus* growth and alleviating inflammation with TCSs and calcineurin inhibitors. Direct strategies target the bacterial load through interventions such as bleach baths, antibiotics, and phototherapy [69,70]. In recent years, bacteriotherapy has emerged as a promising approach for managing AD. Bacteriotherapy achieves its effects by restoring the skin microbial balance (Table 3). In a phase 1 clinical trial, topical application of *Staphylococcus hominis* A9 improved the local EASI and SCORAD scores of patients with AD plus *S. aureus* infection, by inhibiting biofilm formation and supporting skin barrier [71,72]. A phase 2b trial of a topical spray containing the live biotherapeutic B244 demonstrated a 34% reduction in pruritus as measured using the WI-NRS score and amelioration of eczema symptoms in patients with mild to moderate AD. These effects were attributed to the downregulation of Th2 cytokine expression, reduction in *S. aureus* colonization, and anti-inflammatory properties of nitric oxide [73]. Topical omiganan, an antimicrobial peptide, reduced *S. aureus* abundance by 93.5%; however, clinical outcomes were inconsistent. One randomized controlled trial (RCT) reported no significant clinical improvement, whereas another reported modest but statistically significant benefits [74,75]. Topical microbiome transplantation using *Roseomonas mucosa* reduced *S. aureus* load, disease severity, and topical steroid use in small studies. Nonetheless, large trials failed to demonstrate the superiority of this approach over placebo in achieving EASI50 [76,77,78]. Notably, all of the aforementioned studies were conducted over short durations and involved relatively small patient populations (fewer than 1000 participants), underscoring the need for long-term extension studies with larger cohorts to establish the efficacy and safety of bacteriotherapy for AD.

In addition to the skin microbiota, the gut microbiota has received increasing attention from researchers studying AD pathogenesis, particularly because of its involvement in the gut–skin–brain axis. Gut dysbiosis may affect immune responses through several mechanisms, such as Toll-like receptor signaling, the short-chain fatty acid anti-inflammatory axis, and the tryptophan metabolite–aryl hydrocarbon receptor axis [79]. Patients with AD often exhibit limited gut microbiota diversity, characterized by a reduced abundance of beneficial bacteria such as *Bifidobacterium*, and an increased abundance of potential pathogens such as like *Clostridium difficile, Escherichia coli,* and *Bacteroides* [80,81]. Some studies have indicated that disruptions in the gut microbiota are correlated with AD severity and can precede disease onset [82,83,84]. Probiotics and fecal microbiota transplantation have emerged as treatment approaches for AD. Several studies have demonstrated that probiotic formulations containing *Bifidobacterium* and *Lactobacillus* strains can reduce AD severity, improve SCORAD and EASI scores, and enhance gut microbiome composition [85,86,87]. In a small trial involving nine adults with moderate to severe AD, fecal microbiota transplantation resulted in a 77% response rate without any adverse events [88]. Although these early findings are promising, additional research is required to confirm the long-term efficacy and safety of microbiota-based therapies in the management of AD.

### 4.2. The Itch–Scratch Cycle

Chronic itch in AD substantially compromises patients’ quality of life. The underlying mechanism involves a complex interplay between the immune and nervous systems. In patients with AD, hypersensitive skin exhibits exaggerated responses to mild stimuli, whereas repeated scratching disrupts the skin barrier, facilitating allergen penetration and intensifying itch, thereby resulting in a self-perpetuating cycle. Both external stimuli and endogenous pruritogens activate unmyelinated C fibers in the skin, leading to the release of neuropeptides that transmit itch signals to the brain. These neuropeptides also stimulate the release of proinflammatory cytokines, further exacerbating inflammation and perpetuating the itch–scratch cycle [89,90].

#### 4.2.1. IL-31

Although IL-31 is primarily produced by activated Th2 cells in AD, other immune cells, such as dendritic cells, eosinophils, and basophils, also produce this cytokine [91]. IL-31 plays a central role in promoting pruritus in AD by directly activating sensory neurons, stimulating keratinocyte activity, inducing inflammatory mediators release, impairing skin barrier function, and amplifying inflammatory signaling. In addition, IL-31 contributes to epidermal thickening during the chronic Th1-mediated phase [91,92]. Its biological effects are mediated through binding to its receptor, IL-31R, a heterodimer comprising IL-31Rα and the oncostatin M receptor β. This receptor complex is expressed on keratinocytes, unmyelinated C-fiber nerve endings, and neurons in the dorsal horn of the spinal cord [91].

Nemolizumab, a monoclonal antibody targeting IL-31Rα, has been approved in Japan for managing AD-related itch in patients aged ≥ 13 and older. This antibody was recently approved by the US Food and Drug Administration for the treatment of prurigo nodularis. A meta-analysis of six randomized controlled trials—one phase 1, four phase 2, and one phase 3 trial—compared efficacy between nemolizumab (569 patients) and placebo (240 patients) (Table 4). Compared with placebo, nemolizumab considerably mitigated pruritus, leading to an 18.86-point reduction on the pruritus visual analog scale and an 11.76-point reduction in the EASI score with no increase in the rate of adverse events [93]. Long-term data (≥52 weeks) from two phase 3 trials involving Japanese patients aged ≥13 years suggested that nemolizumab, administered at 60 mg every 4 weeks in combination with topical therapies led to sustained improvements in pruritus (66% reduction in pruritus VAS), overall AD symptoms, and quality of life, with a favorable safety profile [94]. In Japanese pediatric patients aged 6–12 years, nemolizumab at a dose of 30 mg every 4 weeks markedly reduced pruritus compared with the effects of placebo. The antibody ensured itch relief by day 2, and its safety profile was consistent with that noted in older patients [95]. Two large international phase 3 trials were conducted across 22 countries among 1728 patients (age ≥ 12 years) with moderate to severe AD. These trials confirmed the efficacy of nemolizumab, with a loading dose of 60 mg followed by 30 mg every 4 weeks resulting in marked improvements in EASI and IGA scores as well as in key secondary outcomes such as pruritus reduction (by week 1) and enhanced sleep quality (by week 16) [96]. Both studies were conducted with background therapy, including TCSs or topical calcineurin inhibitors. Together, the findings indicate that nemolizumab is particularly effective for patients with AD whose pruritus remains inadequately controlled despite topical treatments and antihistamines.

#### 4.2.2. Histamine *4* Receptor (H4R)

Antihistamines targeting histamine H1 receptor (H1R) have exhibited limited efficacy in relieving AD-related pruritus. Histamine, a well-established itch mediator, is primarily released by mast cells and basophils in the skin. It exerts its effects through four receptor subtypes (H1R to histamine H4 receptor [H4R]). Recent clinical research has shifted focus toward the H4R to address the limitations of H1R antagonists (Table 4). Activation of H4R on keratinocytes promotes cellular proliferation and induces TSLP release [104]. Additionally, histamine binding to H4R on Th2 cells increases the production of IL-31, amplifying the pruritic response in patients with AD [105].

Clinical trials evaluating selective H4R antagonists such as JNJ-39758979, ZPL-3893787, and LEO 152020, have yielded mixed results. In a phase 2 trial conducted in Japan, JNJ-39758979 (H4R antagonist) treatment led to only numerical, non-significant improvements in the EASI scores from baseline to week 6. Nonetheless, high-dose treatment markedly improved patients’ pruritus scores. However, the trial was terminated after two participants in the high-dose group developed agranulocytosis, a severe adverse event suspected to be compound-specific and unrelated to H4R inhibition [100]. In another trial, oral ZPL-3893787 (H4R antagonist) treatment resulted in a 50% reduction in the EASI score by week 8 compared with the findings in the placebo group (27% reduction). Clinical improvements with ZPL-3893787 became apparent as early as week 1 or 2 and reached statistical significance by weeks 4 to 6, indicating a rapid onset of anti-inflammatory response. The EASI75 response at week 8 was significantly higher in the ZPL-3893787 group (32%) than in the placebo group (15%; *p* = 0.033). However, no significant difference was observed in mean worst daily pruritus scores between the two groups [98]. LEO 152020, another H4R antagonist, failed to exhibit clinical efficacy in a phase 2 trial [99].

#### 4.2.3. Substance P (SP) and Neurokinin 1 Receptor (NK1R)

Substance P, a neuropeptide from the tachykinin family, is released from the terminals of afferent unmyelinated C-fibers and binds to the neurokinin 1 receptor (NK-1R), which is expressed on endothelial cells, keratinocytes, and mast cells. Activation of the NK-1R by SP triggers several key responses: promoting vasodilation, enhancing the adhesion of leukocytes and monocytes to endothelial cells, stimulating leukotriene B production in keratinocytes, and inducing mast cell degranulation. This degranulation releases itch-inducing mediators, including proteases and histamine, further intensifying the itch response [90,106]. Despite these insights, clinical trials with NK-1R antagonists have been disappointing. Tradipitant, a novel NK-1R antagonist, failed to show efficacy in two Phase 3 trials, and serlopitant, another NK-1R antagonist, was similarly ineffective in a Phase 2 trial [101,103] (Table 4).

## 5. Challenging and Future Perspectives

Current approved therapies primarily target central cytokines of Th2 inflammation, such as IL-4 and IL-13, as well as the JAK/STAT pathway, which mediates downstream signaling for multiple cytokines. While these treatments have proven highly effective, they do not fully address the needs of all patients. In addition, adverse drug reactions which are related to JAK inhibitors, including venous thromboembolism and abnormal lipid profiles, may need further surveillance.

Consequently, the AD therapeutic pipeline is expanding rapidly, new treatments are under investigation aimed at upstream factors like skin microbiomes, alarmins, and the OX40-OX40L pathway, along with various AD endotypes and the itch–scratch cycle. While preclinical studies on these novel treatments have shown promise (Table 5), many have failed to demonstrate efficacy in phase 2 trials. Despite these setbacks, such outcomes provide valuable insights into the disease’s complexity. Trial design considerations, such as the influence of concomitant topical corticosteroid use on placebo effects, the need for longer observation periods for upstream-targeted therapies, and the impact of patient selection based on endotype or disease severity, are all critical to interpreting efficacy results. Nonetheless, some promising developments have emerged. Monoclonal antibodies targeting OX40-OX40L are currently advancing through phase 3 trials, and nemolizumab has shown favorable results in phase 3 studies, indicating potential future additions to the AD treatment landscape.

## 6. Conclusions

This review explored a range of novel therapies for AD, including anti-TSLP, anti-IL-1α, anti-IL-33, anti-IL-36R, anti-IL-12/IL-23, anti-IL-23, and anti-IL-17, as well as histamine receptor antagonists and NK-1 receptor antagonists. Unfortunately, these therapies did not meet efficacy goals in phase 2 or 3 trials. However, fezakinumab (anti-IL-22) showed promise in patients with severe AD and high baseline IL-22 levels in subgroup analyses, while other novel treatments, such as amlitelimab (anti-OX40L), rocatinlimab and telazorlimab (anti-OX40), and nemolizumab (anti-IL-31R), yielded encouraging results. Collectively, these findings highlight the expanding AD therapeutic landscape and offer hope for more targeted and effective treatments that can address diverse disease pathways and patient subtype.

## Figures and Tables

**Table 3:** Clinical trials of novel treatments targeting modulation of skin microbiome for atopic dermatitis.

Target	Medication	Study Design	Primary Outcome	Clinical Trial	Status of Investigation
Antibacterial activity	Topical *staphylococcus hominis* A9	54 patients with AD with positive *S. aureus* culture colonized lesion	Per-participant daily event rate of treatment-emergent adverse event targeted microbiome transplant: 0.19 (95% CI: 0.12 to 0.29) Placebo: 0.34 (95% CI: 0.20 to 0.58) *p* = 0.075	NCT03151148 (Phase I) [72]	Complete
	Topical biotherapeutic B244	547 patients with AD with mild to moderate pruritus	Mean change in WI-NRS at Week 4 B244 O.D. 5.0: −2.8 (0.184) (*p* = 0.0148) B244 O.D. 20.0: −2.8 (0.184)(*p* = 0.0143) Placebo: −2.1 (0.180)	NCT04490109 (Phase II) [73]	Complete
	Topical omiganan	37 adults with AD	Change in local objective SCORAD index at Week 4 Omiganan 2.5%: −18.5% (95% CI: −32.9% to −1.0%) (*p* = 0.04) Omiganan 1%: −13.4% (95% CI: −28.4% to 4.6%) (*p* = 0.13)	NCT02456480 (Phase II) [75]	Complete
		80 adults with mild to moderate AD	Abundance of Staphylococcus at Week 4 Omiganan 1%: −15.1 (95% CI, −28.6 to −1.7) (*p* = 0.03) Omiganan 2.5%: −17.2 (95% CI, −30.4 to −4.1) (*p* = 0.01) Change in local objective SCORAD index at Week 4 Omiganan 1%: 2.0 (95% CI: 0.52 to 4.51) (*p* = 0.12) Omiganan 2.5%: 2.52 (95% CI: 0.0 to 5.04) (*p* = 0.05)	NCT03091426 (Phase II) [74]	Complete
	Topical *Roseomonas mucosa* (FB-401)	154 patients (age ≥ 2 years) with mild to moderate AD	EASI 50 at Week 16 FB−401 3 times per week: 57.9% (*p* = 0.7567) Placebo: 60.3%	NCT04504279 (Phase II) [76]	Complete, but failed to meet efficacy

**Table 4:** Clinical trials of novel treatments targeting itch–scratch cycle for atopic dermatitis.

Target	Medication	Study Design	Primary Outcome	Clinical Trial	Status of Investigation
IL-31RA	Nemolizumab	143 Japanese (age ≥ 13 years) with AD and moderate-to-severe pruritus	% change in the VAS score for pruritus at week 16 Nemolizumab 60 mg q4w: −42.8% Placebo: −21.4% least-squares mean difference between two groups: −21.5% (95% CI: −30.2 to −12.7; *p* < 0.001)	JapicCTI-173740 [97]	Complete
		89 Japanese patients (age ≥ 6 and < 13 years) with AD and moderate-to-severe pruritus	Change in 5-level itch score from baseline at week 16 Nemolizumab 30 mg q4w: −1.3 Placebo: −0.5 Least-squares mean difference between two groups: −0.8 (95% CI: −1.1 to −0.5; *p* < 0.0001)	Japan Registry for Clinical Trials 2080225289 [95]	Complete
		941 patients (age ≥ 12 years) with moderate to severe AD	IGA clear or almost clear and at least a 2-grade improvement from baseline at Week 16 Nemolizumab loading 60 mg/30 mg q4w: 35.6% (*p* = 0.0003) Placebo: 24.6% EASI75 at Week 16 Nemolizumab loading 60 mg/30 mg q4w: 43.5% (*p* < 0.0001) Placebo: 29.0%	NCT03985943 (Phase III) [96]	Complete
		787 patients (age ≥ 12 years) with moderate to severe AD	IGA clear or almost clear and at least a 2-grade improvement from baseline at Week 16 Nemolizumab loading 60 mg/30 mg q4w: 37.7% (*p* = 0.0006) Placebo: 26.0% EASI75 at Week 16 Nemolizumab loading 60 mg/30 mg q4w: 42.1% (*p* = 0.0006) Placebo: 30.2%	NCT03989349 (Phase III) [96]	Complete
H4R	ZPL-3893787/ Adriforant	98 adults with moderate to severe AD	Reduction of WI-NRS at week 8 Adriforant 30 mg QD: −3.03 (2.186) (*p* = 0.249) Placebo: −2.66 (2.057)	NCT02424253 (Phase II) [98]	Complete but failed to meet efficacy
	LEO 152020	216 adults with AD	Change in the EASI score at Week 16 LEO 152020(Higher Dose): −9.99 (95% CI: −12.85 to −7.13) LEO 152020(Middle Dose): −8.83 (95% CI: −12.63 to −5.04) LEO 152020(Lower Dose): −8.87 (95% CI: −12.47 to −5.28) Placebo: −9.11(95% CI: −11.88 to −6.35)	NCT05117060 (Phase II) [99]	Complete but failed to meet efficacy
	JNJ 39758979	88 Japanese adults with moderate AD	Changes in EASI scores at Week 6 JNJ−39758979 100 mg: median, −3.70 (*p* = 0.1672) JNJ−39758979 300 mg: median, −3.00 (*p* = 0.1992) placebo: median, −1.30	NCT01497119 (Phase II) [100]	Terminate due to 2 cases of agranulocytosis
NK1R	Tradipitant	375 adults with AD suffering from chronic pruritus	Reduction of WI-NRS at week 8 Mean (Standard Deviation) Oral tradipitant BID: −3.6 (2.8) Placebo: −3.5 (2.75	NCT03568331 (Phase III) [101]	Complete but failed to meet efficacy
		87 adults with AD suffering from chronic pruritus	WI-NRS responder rate at week 2 (achieve at least 4 points reduction from baseline) Oral tradipitant BID: 4.8% Placebo: 9.3%	NCT04140695 (Phase III) [102]	Terminate
	Serlopitant	484 patients (age ≥ 13 years) AD with pruritus	Reduction of WI-NRS at week 6 Oral serlopitant loading 15 mg/5 mg QD: −2.25 (2.198) Oral serlopitant loading 3 mg/1 mg QD: −2.32 (2.418) Placebo: −2.01 (2.212)	NCT02975206 (Phase II) [103]	Complete but failed to meet efficacy

**Table 5:** Novel therapeutic strategies categorized by mainly target symptoms of atopic dermatitis.

Target Symptom	Therapeutic Strategy	Example
Skin Inflammation	- topical JAK inhibitor - oral JAK inhibitor - IL-4 inhibitor - IL-13 inhibitor - OX40/OX40L inhibitor - IL-17 inhibitor (limited efficacy) - IL-22 inhibitor - IL-23 inhibitor (limited efficacy)	- Delgocitinib, ruxolitinib - Upadacitinib, abrocitinib, baricitinib - Dupilumab - Tralokinumab, lebrikizumab - Rocatinlimab, telazorlimab, amlitelimab - Secukinumab - Fezakinumab - Ustekinumab, risankizumab
Itch	- IL-31 inhibitor - Histamine 4 receptor (limited efficacy) - neurokinin 1 receptor (limited efficacy)	- Nemolizumab - Adriforant, LEO 152020, JNJ 39758979 - Tradipitant, serlopitant
Secondary Infection/Colonization	- bacteriotherapy	- Topical Staphylococcus hominis A9, bio-therapeutic B244, omiganan

## Data Availability

No new data were created.

## Abbreviations

The following abbreviations are used in this manuscript:

AD	atopic dermatitis
Th2	T helper 2
IL	interleukin
JAK/STAT	Janus kinase/signal transducer and activator of transcription
TSLP	thymic stromal lymphopoietin
EASI	eczema area and severity index
POEM	patient-oriented eczema measure
DLQI	dermatology life quality index
PP-NRS	peak pruritus numerical rating scale
TCS	topical corticosteroids
RCT	randomized controlled trial
H4R	Histamine 4 Receptor
IGA	Investigator’s Global Assessment
NK1R	neurokinin 1 receptor
SP	Substance P
*SCORAD*	SCORing Atopic Dermatitis
*TSLP*	thymic stromal lymphopoietin
*WI-NRS*	worst itch numeric rating scale
